# The Influence of SiC and Al_2_O_3_ Particles on the Microstructure and Tribological Properties of the EN-GJL-150 Cast Iron-Based Composite

**DOI:** 10.3390/ma19102040

**Published:** 2026-05-13

**Authors:** Jaroslaw Piatkowski, Mateusz Wojciechowski, Tomasz Matula, Katarzyna Nowinska

**Affiliations:** 1 Materials Innovation Laboratory, Faculty of Materials Science and Industrial Digitalization, Silesian University of Technology, Krasinskiego 8, 40-019 Katowice, Poland; jaroslaw.piatkowski@polsl.pl; 2 Independent Researcher, 43-600 Jaworzno, Poland; mateu.wojciechowski@gmail.com; 3 Department of Metallurgy and Recycling, Faculty of Materials Science and Industrial Digitalization, Silesian University of Technology, Krasinskiego 8, 40-019 Katowice, Poland; tomasz.matula@polsl.pl; 4 Department of Applied Geology, Faculty of Mining, Safety Engineering and Industrial Automation, Silesian University of Technology, ul. Akademicka 2, 41-100 Gliwice, Poland

**Keywords:** EN-GJL-150 cast iron, SiC and Al_2_O_3_ reinforcing particles, cast iron microstructure, graphite nucleation

## Abstract

This article presents preliminary research on the development of a cast iron–ceramic composite for modern braking systems, such as brake discs. The composite matrix is gray cast iron with flake graphite (EN-GJL-150). The reinforcing phase is a porous ceramic composed of SiC and Al_2_O_3_ particles introduced separately (10% each) and together (70% SiC + 30% Al_2_O_3_). These particles were applied as a suspension onto polyurethane foam, yielding a ceramic structure with a pore density of up to 10 ppi. The resulting insert was placed in a mold cavity, and cast iron was poured into it. The resulting samples were treated as brake disc material, with a pad made of the commercial friction material P50094 serving as the countersample. Tribological tests showed that the lowest sample wear (average 2.23 mg/5000 m) was achieved for the composite reinforced with SiC + Al_2_O_3_ particles. This is probably due to the synergy between the antifriction properties of these particles and the lower friction coefficient (µ = 0.180–0.22). Similar mass loss values and the smallest difference between the tested samples were observed for composites with SiC particles (3.01 mg/5000 m) and Al_2_O_3_ (3.30 mg/5000 m). The second part consisted of microstructural studies. Microstructural analysis of the EN-GJL-150 + SiC + Al_2_O_3_ composite revealed a previously unobserved nucleation phenomenon at the cast iron–ceramic interface. This confirmed the general assumptions of Riposan’s theory regarding the involvement of oxide microinclusions and complex manganese sulfides of the (Mn, X)S type in the nucleation and crystallization of graphite precipitates. It was also found that, in the case of “in situ” GJL-150 + SiC + Al_2_O_3_ composites, this theory should account for the beneficial role of ceramic particles in promoting the uniform distribution of type A graphite flakes, which nucleate on their surfaces in the transition zone. Thus, the nucleating role of oxide microinclusions (the first stage of Riposan’s theory) could be taken over by SiC and Al_2_O_3_ particles, constituting a substrate for the heterogeneous nucleation of (Mn, X)S sulfides.

## 1. Introduction

One of the most important parts of every vehicle is the braking system, as it affects the safety of its occupants and other road users. A brake is a mechanical device that absorbs the kinetic energy of a rotating wheel and converts it into heat through friction between the brake pads and the brake disc. During braking, the brake shoe contacts the outer friction surface, and contact with the rotating drum slows the wheel [1,2]. To ensure braking effectiveness in all weather conditions, better braking force control (shortening braking distance), and effective heat dissipation (avoiding brake fade due to overheating), various design changes are introduced to the disc [3,4,5]. This must be accompanied by material innovations that reduce the disc’s weight and increase its durability and corrosion resistance [6,7]. High Yang modulus and high resistance to abrasive wear are also crucial criteria for selecting brake disc materials, ensuring longer brake life [8,9,10]. The cost of manufacturing brake discs is also important and, based on production optimization, should be as low as possible.

One way to reconcile these seemingly contradictory criteria is to use gray cast iron. This is due to its many advantages, including good casting properties (low shrinkage, good castability), ease of machining, high abrasion resistance (due to graphite’s lubricating properties), vibration absorption, and an affordable price [11,12]. To maintain market competitiveness and meet growing customer demand, the production process for cast iron castings is continually being developed. This primarily concerns the controlled graphite nucleation process. Its morphology and matrix type, combined with its chemical composition, determine the mechanical properties [13,14,15], which can be further improved through heat treatment [16,17]. In cast iron, elements that support graphite nucleation include, for example, silicon, manganese, and trace amounts of calcium, aluminum, strontium, barium, and zirconium [18,19,20]. Their absence or insufficient quantity causes cast iron to crystallize (partially or completely) metastably [21,22]. The role of these additives is to enable the formation of numerous particles (so-called heterogeneous substrates) that favor graphite formation. This ensures constant conditions during the nucleation and growth of graphite flakes, leading to high, repeatable performance properties, especially hardness, in cast iron castings.

The mechanism of graphite nucleation in gray cast irons is described by various hypotheses [23,24], one of the most well-known being the Riposan theory [25,26]. In simple terms, it assumes a three-stage model of graphite formation:

1. Formation of oxides (usually up to 2 μm in diameter) in the alloy, based on strongly deoxidizing elements (e.g., aluminum and zirconium);
2. Crystallization of complex (Mn, X)S sulfides (usually up to about 5 μm in size), which nucleate on the oxides from the first stage;
3. Interaction of modifying elements (e.g., calcium, strontium), which improve the ability of (Mn, X)S sulfides to nucleate graphite due to their good crystallographic match with graphite.

Research results [25,27] indicate that such a model of graphite nucleation requires the introduction of strong “deoxidizers” into liquid cast iron, which create numerous very small microinclusions. Furthermore, the presence of sulfur (up to approximately 0.07 wt.%) is necessary for the crystallization of complex sulfides of the (Mn, X)S type and residual aluminum (0.001–0.003 wt.%) [26]. This allows for a reduced degree of undercooling ∆T and an appropriate number of eutectic cells in the modified cast irons. Furthermore, a lower ∆T favors the crystallization of a favorable eutectic with randomly oriented, uniformly distributed “A” type graphite flakes [26].

This theory, although interesting, does not address the solution strengthening of cast iron with other additives, e.g., ceramic particles. Some studies, e.g., [28,29], suggest that phenomena occurring during cast iron crystallization could combine a ferritic-pearlitic matrix with advanced technical ceramics. The possibility of producing multiphase iron-based materials is one of the most interesting challenges in contemporary materials engineering. The interaction between liquid cast iron and ceramic particles, e.g., titanium carbide (TiC) [30], silicon carbide (SiC), zirconium oxide (ZrO_2_) [31], or aluminum oxide (Al_2_O_3_) [32], results in a combination of favorable physicochemical properties of cast iron and ceramics. This primarily concerns low wettability, small differences in thermal expansion and conductivity, and a high vibration-damping coefficient [28]. Unfortunately, many of these studies do not explain the mechanism of graphite nucleus formation on the ceramic surface or the method for introducing reinforcement (in the form of particles or fibers) into the matrix, both of which are crucial for achieving optimal strength properties [33].

Therefore, a more thorough understanding of graphite nucleation in gray cast irons with ceramic particles as a reinforcing phase is crucial. This need stems primarily from the desire to improve the performance of iron castings, such as those used in braking systems. The controlled microstructure of such castings ensures that the products can absorb and rapidly dissipate high levels of kinetic energy while meeting stringent environmental standards. Non-exhaust emissions, primarily from brake and tire wear and tear, will soon be subject to mandatory limits. The introduction of particulate matter (PM) emission limits, as mandated by the European Parliament’s regulations under the Euro7 standard [34], requires changes to braking system design and materials. This requirement applies, among others, to gray cast irons, in which the microstructure determines the required performance properties.

One of them is abrasion resistance, considered the main criterion for the use of cast iron in industry [18,35], especially in wheeled transport [11]. Many studies, e.g., [36], indicate that the durability and reliability of devices are determined by resistance to tribological wear, lubrication, and the energy input required to overcome frictional resistance. A significant role in achieving this goal is played by the surface layers of moving machine elements [37,38] and multiphase composites based on cast iron [36].

Regardless of the technology used to produce abrasion-resistant materials, the rationale behind this new research is to improve the performance of tribological systems while meeting stringent environmental standards. Modern braking systems, therefore, require components capable of absorbing and rapidly dissipating high levels of kinetic energy while maintaining structural stability. Given the aforementioned need to reduce particulate emissions, future materials for braking systems must exhibit greater resistance to abrasive wear than those currently used.

According to the authors of this publication, further research is needed on the design of new brake disc materials, whose appropriate combinations of structural components will enable high tribological resistance.

Taking the above into account, the research aimed to develop a composite based on EN-GJL-150 cast iron reinforced with SiC and Al_2_O_3_ ceramic particles (separately and in combination), and to analyze the microstructure and its effect on tribological properties. The results obtained were compared with those of cast iron without ceramic particles. To achieve this goal, the scope of the research included:

- Development of a composite concept: EN-GJL-150 gray cast iron—SiC + Al_2_O_3_ particles.
- Melting and casting of a cast iron–ceramic composite. Measurement of chemical composition.
- Tribological studies of three friction pairs: GJL-150 cast iron + SiC; GJL-150 cast iron + Al_2_O_3_; GJL-150 cast iron + SiC + Al_2_O_3_ and comparison with GJL-150 cast iron without ceramic particles.
- Macro- and microstructure studies.
- Proposing a diagram of graphite nucleation in cast iron composites—verification of Riposan’s theory.

## 2. Materials and Methods

### 2.1. Methodology for Making Melts and Chemical Composition

Commercial EN-GJL-150 cast iron, Brembo, Bergamo, Italy used for castings such as brake discs, was selected for testing. The cast iron, modified with Elkem SuperSeed (0.15–0.25%), was cast from 1440 ± 5 °C into a 300 × 300 mm sand mold with a 150 × 100 × 20 mm cavity. A porous insert with an open porosity of 10 ppi (pores per inch) was placed in the center of the molding box. The ceramic insert with a foam structure was made by repeatedly coating polyurethane foam with a slurry based on SiC and Al_2_O_3_ particles (separately and in combination). To bond the ceramic particles into a uniform porous structure, the entire assembly was fired at 1000 °C. During the pouring of liquid cast iron, the insert’s function is to form a composite structure of cast iron and ceramic particles. Three variants of cast iron–ceramic castings were made, in which the reinforcing phase is ceramic foam, containing:

1. GJL-150 cast iron + 10% SiC;
2. GJL-150 cast iron + 10% Al_2_O_3_;
3. GJL-150 cast iron + SiC + Al_2_O_3_.

The content of SiC and Al_2_O_3_ reinforcing particles, and their combination with the activated porous surface, were selected based on industrial practice [39] and research [40]. The method of making ceramic inserts was presented in [41]. Research [40,41] showed that the optimal particle content is 10 wt.% SiC; 10 wt.% Al_2_O_3_ (introduced separately) and (70 wt.% SiC and 30 wt.% Al_2_O_3_) introduced together.

After pouring the mold, samples were taken for chemical composition analysis. Tests were performed using a spark optical emission spectrometer for quantitative elemental analysis of solid metal samples cast into a whitened die (Spectro LAB S—Ametek, Kleve, Germany). Ten measurements were taken, and the arithmetic mean was calculated. Results were rounded to two decimal places. During the production of prototype composite brake discs, the chemical composition was measured twice: once after determining the transition grade of cast iron in the melting furnace, and again after pouring the cast iron into the pouring furnace. Each time, two samples were taken from the liquid cast iron at 1400 °C, poured into a copper die to “whiten” the structure and precipitate carbon as carbides. The sample for chemical composition analysis was roughly ground from the spark exciter side of the spectrometer.

### 2.2. Tribological Research Methodology

From these prepared castings, samples were cut with a diameter (D = 45 mm), thickness (H = 5 mm), and surface roughness (Ra = 1.8 µm). The counter-samples were made of commercial friction material P50 094 Brembo, Bergamo, Italy. This is a typical material for PEX brake pads, Brembo, Bergamo, Italy complies with OEM standards, and ECE-R90 homologation [42] Brembo P 50 094 brake pads, part of the Premium Prime line, feature Low-Metallic friction material. The chemical composition depends on the specific type (organic, ceramic, metallic). It typically includes a mixture of friction materials (aluminum), fillers and binders (synthetic resins), and metal filings (copper, bronze) to improve heat conduction, enable high-temperature braking, and ensure good performance and durability. The brake pads were cut and turned to match the pins, with a diameter d = 5 mm and a height h = 20 mm. The OE-spec pad material, Brembo, Bergamo, Italyhas been developed to provide a stable, equivalent coefficient of friction to standard brakes. The casts prepared in this way and cleaned with spirit served as samples for tribological tests conducted at room temperature under dry-friction conditions. To ensure repeatable friction conditions and eliminate the influence of random surface factors, the counter-sample was reconditioned each time. This process involved cleaning the counter-sample surface and degreasing it with spirit.

Abrasion tests were performed on a T-01M tribological tester, Institute of Exploitation Technology, Radom, PolandThis is a pin-on-disc device, Brembo, Bergamo, Italy for testing the friction and wear of structural materials (Institute of Exploitation Technology, Radom, Poland). Five tests were performed for each abrasive pair, using the following tribological parameters:

- Sliding radius r = 14 mm;
- Friction distance s = 5000 m, which corresponds to 100 braking cycles of a 2000 kg car in city traffic;
- Unit pressure p1 = 1 MPa;
- Sliding speed v = 0.5 m s^−1^ under friction and wear conditions, which corresponds to braking the vehicle at the minimum permissible speed in the city center of 30 km h^−1^.

The mass losses of the brake disc material sample (ΔmGJL), Brembo, Bergamo, Italy and the mass of the friction material pin (ΔmP), Brembo, Bergamo, Italy were determined using an A&D HM300 analytical laboratory balance, Mettler-Toledo, Columbus, USA which allows measurements up to 300 g with an accuracy of ±0.1 mg. The samples were weighed four times and cleaned with spirit. The samples were stored in a room with constant temperature and humidity. During the tests, friction forces were measured and recorded using strain-gauge force transducers coupled to a Spider 8 strain-gauge bridge, Institute of Exploitation Technology, Radom, Poland with a mean-square error of up to 3%.

The tests were conducted under conditions simulating driving a passenger car in city traffic at the lowest permissible speed of 30 km/h. The assumed speed corresponds to the wear friction conditions in tribological tests of 0.5 m/s. The friction distance corresponds to 100 braking cycles of a 2000 kg vehicle in city traffic. Constant dry friction conditions were assumed. In each case, the friction system is considered unrun-in (factory new).

### 2.3. Methodology for Performing Microstructure Tests

Microstructural examination of LM Light Microscope) was performed using a Leica MEF-4M optical microscope (Leica Microsystems GmbH, Wetzlar, Germany) and a Keyence VHX-X1 digital microscope (Keyence International, Mechelen, Belgium). SEM (Scanning Electron Microscope) examinations were performed using a Tescan Mira scanning electron microscope (Tescan Brno, Brno, Czech Republic). The examinations were performed on polished and nital-etched and Stead’s reagent-etched microsections.

**Figure 1:**
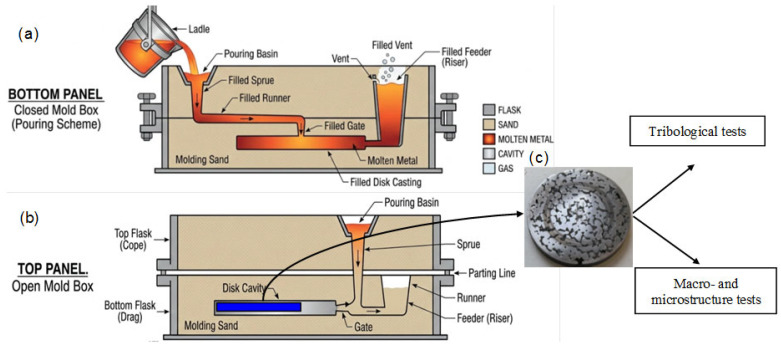
Schematic diagram of the process of casting cast iron into a mold containing a porous insert with ceramic particles: (**a**) beginning; (**b**) final stage; (**c**) cutting location and appearance of test samples.

Ten photographs were taken; of these, the selected images represent the microstructure of the tested cast iron, including ceramic particles. The diagram and sampling location for the tests are shown in Figure 1.

## 3. Results

The chemical composition of cast iron is presented in Table 1.

### 3.1. Tribological Test Results

**Figure 2:**
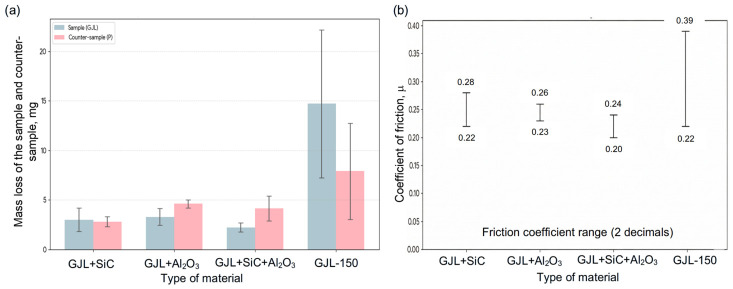
Change in (**a**) mass loss of the sample and counter-sample; (**b**) friction coefficient with error bars.

**Figure 3:**
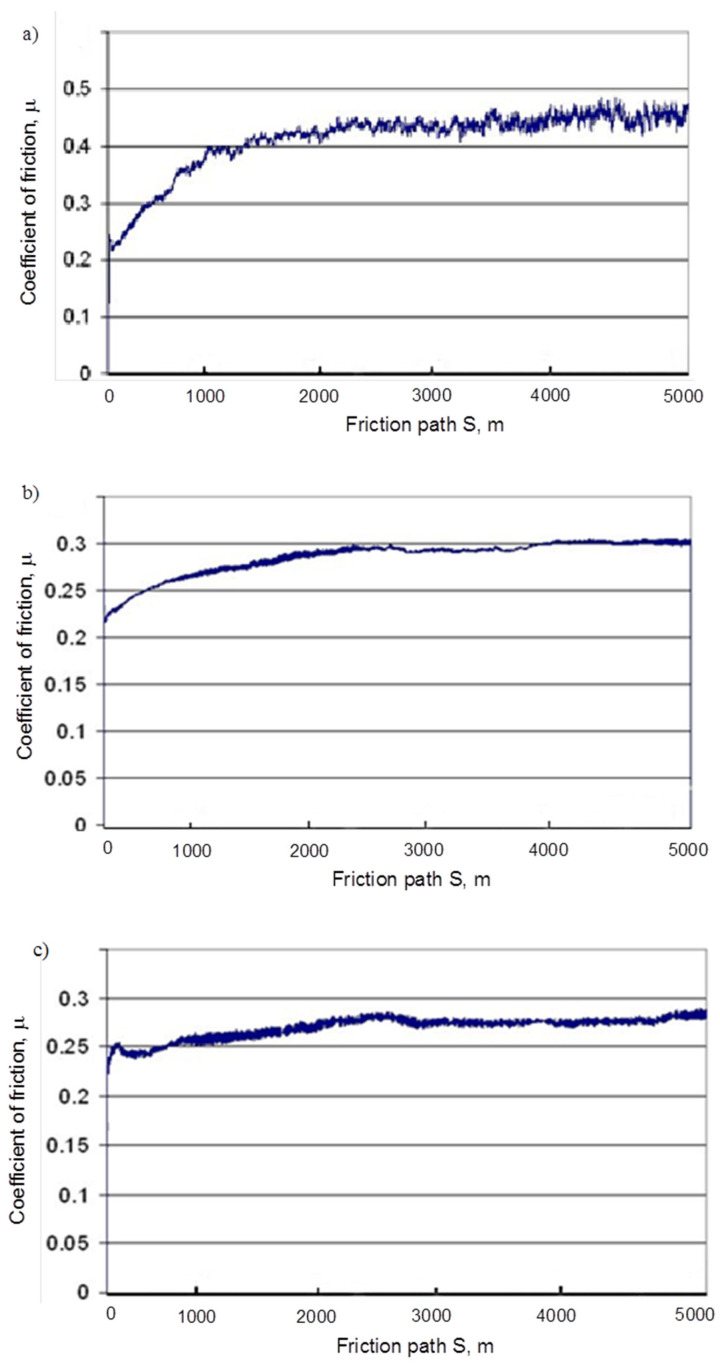

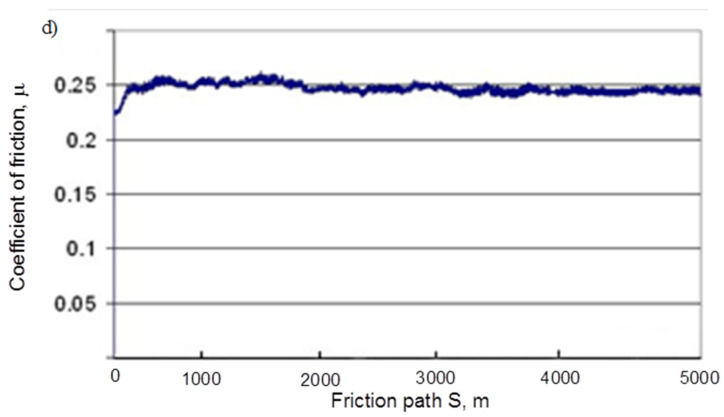
Example graphs of the change in the friction coefficient as a function of the friction path for the following combinations: (**a**) cast iron EN-GJL-150; (**b**) EN-GJL-150 + SiC; (**c**) EN-GJL-150 + Al_2_O_3_; (**d**) EN-GJL-150 + SiC + Al_2_O_3_—counter-sample friction material P50 094.

The results of tribological tests (five tests for each material) of the EN-GJL-150 + SiC, EN-GJL-150 + Al_2_O_3_, and EN-GJL-150 + SiC + Al_2_O_3_ composites and EN-GJL-150 cast iron are presented in Table 2, Table 3 and Table 4. The average mass loss of the samples, the counter-sample, and the coefficient of friction, together with the error bars, are shown in Figure 2. The course of the change in the coefficient of friction for all friction pairs is shown in Figure 3.

Since only five tests were performed in Table 2, Table 3 and Table 4 (for each friction pairing) and the results are quite divergent, they should be verified for statistical significance. The presented results were verified to be consistent with a normal distribution, and the variances for the tested composites (excluding pure EN-GJL-150 cast iron) were equal (a necessary condition). Therefore, a t-Student test for small groups (*n* < 30) was performed. The standard significance level of α = 0.05 was adopted. Based on the results obtained, the dispersion of the results presented in Table 2, Table 3 and Table 4 is below the assumed acceptable confidence level. It should be noted that a comparison was made between the means of the developed composites (e.g., EN-GJL-150 + SiC cast iron) and the countersample. Considering the frictional association of both materials (populations of samples and counter-samples, which in this case is not the essence of the research), the confidence level is at the limit of the α = 0.0525.

### 3.2. Results of Macro- and Microstructure Tests

**Figure 4:**
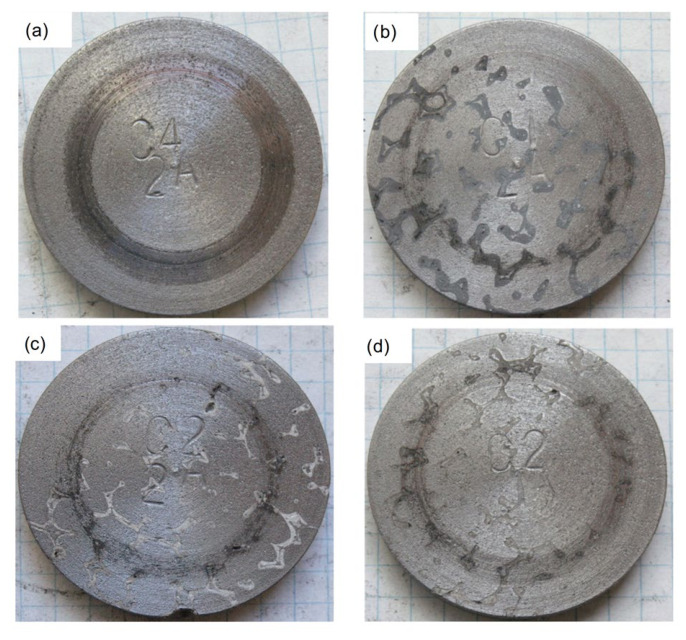
Macrostructure of samples after tribological tests: (**a**) cast iron EN-GJL-150; (**b**) composite EN-GJL-150 + SiC; (**c**) EN-GJL-150 + Al_2_O_3_; (**d**) EN-GJL-150 + SiC+ Al_2_O_3_ after tribological tests.

Examples of macrostructures of GJL-150 cast iron and GJL-150 + SiC, GJL-150 + Al_2_O_3_, and GJL-150 + SiC + Al_2_O_3_ composites are shown in Figure 4.

**Figure 5:**
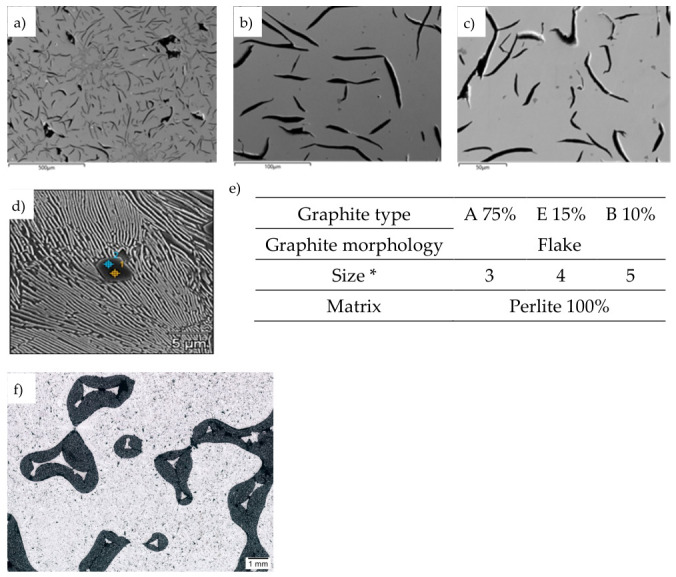
Representative microstructures of LM cast iron (**a**–**c**), pearlitic matrix (**d**), quantitative analysis (**e**), and cast iron—SiC + Al_2_O_3_ particles composite—unetched sample (**f**). * based on the standard PN-EN ISO 945-1 Microstructure of cast iron—Part 1: Classification of graphite precipitations based on visual analysis) [43].

**Figure 6:**
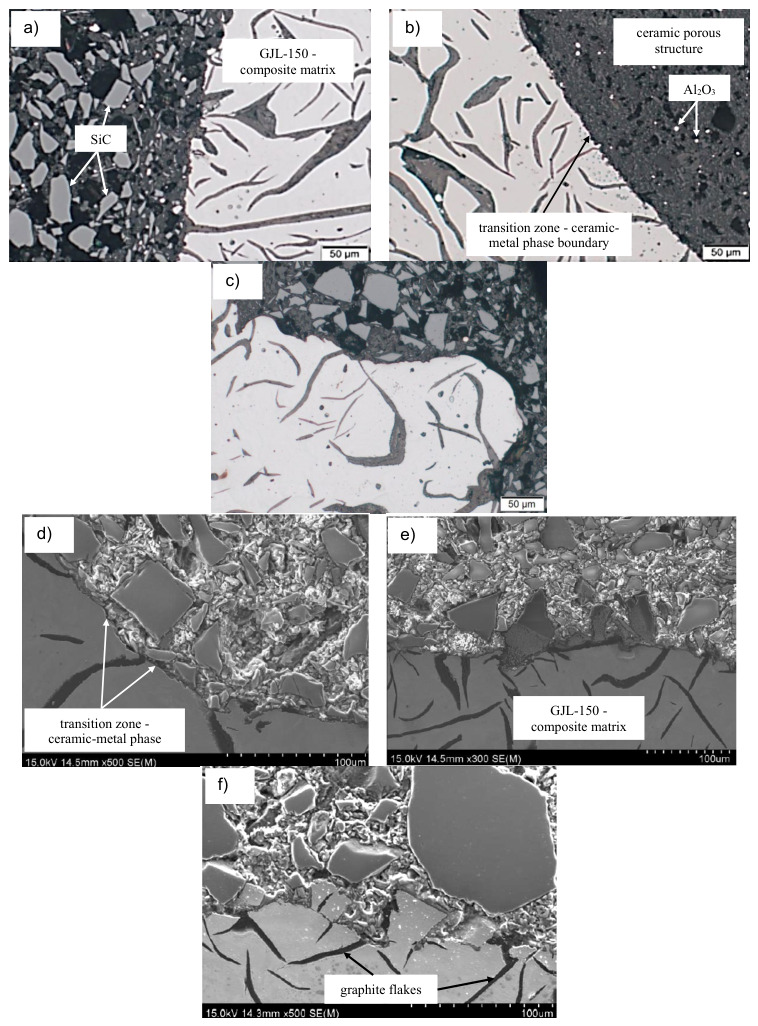
Microstructure of the cast iron–ceramics joint area with visible interface between the matrix and porous structure: (**a**) EN-GJL-150 + SiC; (**b**) EN-GJL-150 + Al_2_O_3_; (**c**) EN-GJL-150 + SiC+ Al_2_O_3_; (**a**–**c**) LM; (**d**–**f**) SEM.

**Figure 7:**
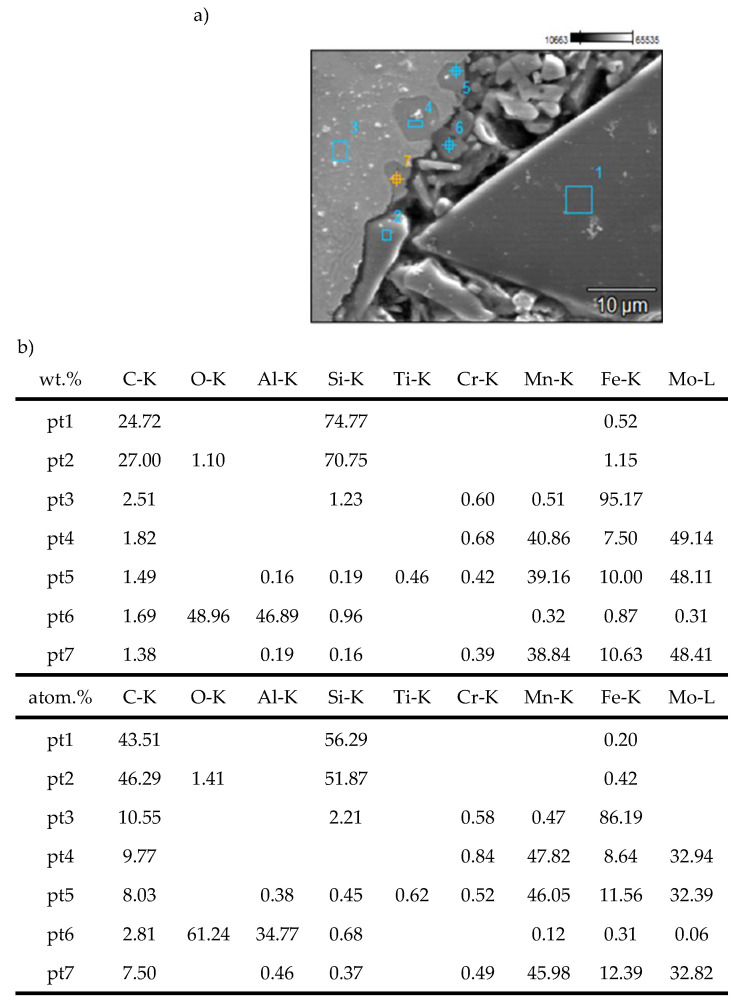
SEM microstructure of the EN-GJL-150 + SiC + Al_2_O_3_ composite (**a**) and the results of chemical composition microanalysis (**b**) at points 1 and 2.

**Figure 8:**
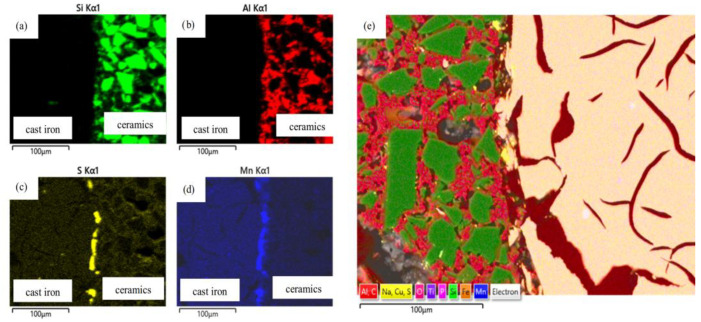
Maps of relative concentration of EDS (Energy-dispersive X-ray spectroscopy) elements at the cast iron–ceramic interface: (**a**–**e**) silicon carbide—component of porous ceramics SiC + Al_2_O_3_; (**b**–**e**) sulfur—component (Mn, X)S; (**c**–**e**) manganese—component (Mn, X)S; (**a**–**c**) for the ENGJL-150+ SiC + Al_2_O_3_ composite; (**d**) for the ENGJL-150 + SiC composite.

Representative microstructures of the EN-GJL-150 composite with SiC and Al_2_O_3_ particles are shown in Figure 5, Figure 6, Figure 7 and Figure 8.

**Figure 9:**
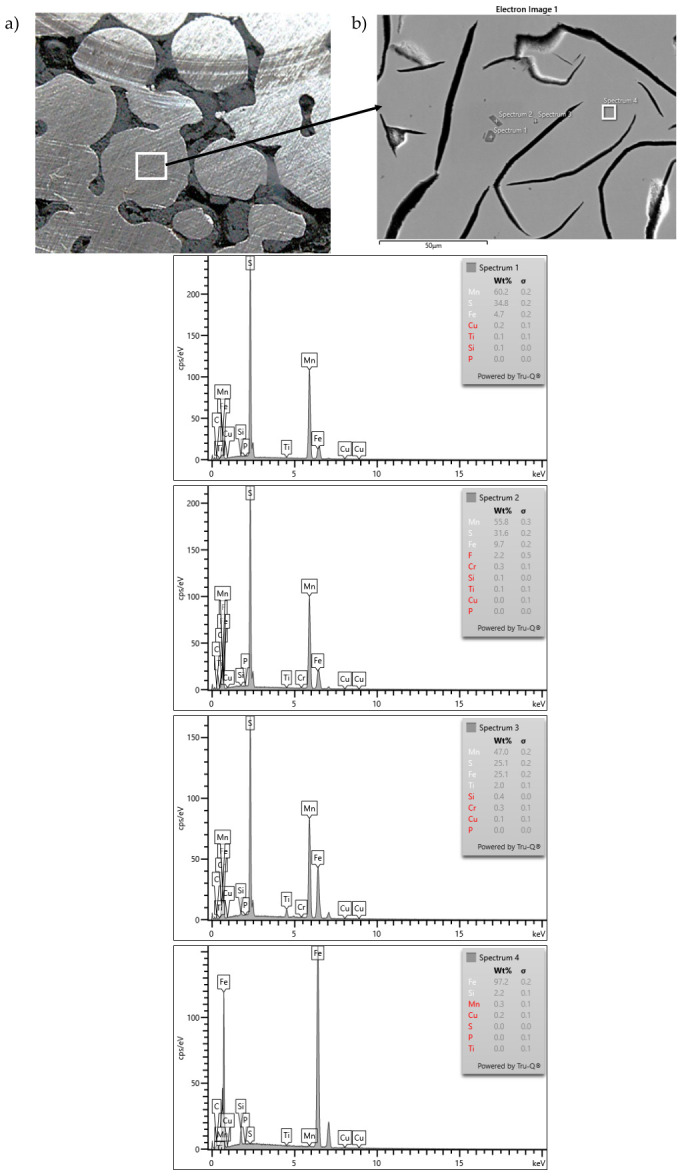
Macro- (**a**) and microstructure (**b**) of the EN-GJL-150+ SiC + Al_2_O_3_ composite and the results of EDS microanalysis at points 1 to 4.

The microstructure of the worn surfaces of the samples after tribological tests is shown in Figure 9.

## 4. Discussion

The reason for undertaking this research was the need to identify alternative materials with tribological properties comparable to or better than those of the previously used grey cast iron EN-GJL-150, assuming the matrix would be grey cast iron. This assumption stems from the need to meet the required mechanical properties and to optimize production costs.

Therefore, a concept was developed to produce a composite of GJL-150 cast iron reinforced with ceramic particles. This method involved placing a porous ceramic insert in the mold cavity, filling the casting volume by at least 10%.

The reinforcement used in the study is a porous “ceramic foam” structure with a pore density of 10 PPI (pores per inch), corresponding to a large, open-cell structure that facilitates the free flow of liquid cast iron, minimizing hydraulic resistance during insert filling. The chemical composition of the reinforcement, 70 wt.% SiC + 30 wt.% Al_2_O_3_, determines a series of complex reactions at the metal-ceramic interface. Three series of cast iron–ceramic castings (representing a brake disc) were made, containing EN-GJL-150 gray cast iron reinforced with particles:

1. SiC;
2. Al_2_O_3_;
3. SiC and Al_2_O_3_.

The counter-sample was a brake pad made of commercial friction material, type P50 094, PEX (Brembo’s trade name for the material).

The combination of the matrix and the strengthening phase allows for increased mechanical and tribological properties and better heat dissipation (thermal conductivity of cast iron λ = 48–60 W (m K)^−1^, while SiC is approx. 120–360 W (m K)^−1^) while maintaining the price at a level similar to the traditional method of smelting cast iron.

The first part of the research concerned selected tribological properties, i.e., mass loss of the cast iron sample (∆mGJL), mass loss of the counter-sample (∆mP), and friction coefficient (µ), as well as the developed composites and comparison with EN-GJL-150 cast iron without reinforcing particles (Table 2, Table 3 and Table 4).

Figure 2a shows the results of tribological mass-loss tests for the tested frictional combinations. The mass loss of the samples and counter-samples across all tested combinations is much lower than that of pure cast iron. The wear of the EN-GJL-150 + SiC combination is low, maintaining the required sample-to-counter-sample wear relationship. For the EN-GJL-150 + Al_2_O_3_ and EN-GJL-150 + SiC + Al_2_O_3_ composites, a wear-like change occurred (the counter-sample wore out faster than the sample). The developed composites were also characterized by a lower coefficient of friction (μ) and greater stability (repeatability of results ranging from 0.18 to 0.22) compared to pure cast iron (0.22 to 0.46; Table 4).

Analysis of the data presented in Table 2, Table 3 and Table 4 and the friction coefficient recording graphs indicates a gradual increase with friction distance (S). For GJL-150 samples with ceramic particles (Figure 3b–d), this increase is much smaller, or even negligible, than for cast iron alone (Figure 3a). A higher value of the friction coefficient provides better adhesion and prevents slippage (e.g., in tires, brakes, etc.). It should also be noted that the machined samples were not fully covered with a porous structure. The coefficient values of µ = 0.3 and µ = 0.4 required in the automotive industry [39] were achieved only during grinding of standard GJL-150 cast iron (without ceramic particles).

The lowest disc wear (2.23 mg/5000 m) was obtained for samples containing SiC + Al_2_O_3_ particles. This is likely the result of the synergy of the antifriction properties of these particles and the lower friction coefficient (µ = 0.18–0.22). Similar mass-loss values and the smallest difference between the tested samples were observed for porous inserts containing separate SiC particles (3.01 mg/5000 m) and Al_2_O_3_ (3.30 mg/5000 m). However, it should be noted that the wear of the countersample was higher than that of the tested sample for Al_2_O_3_ alone and SiC + Al_2_O_3_.

The results of the tribological tests indicate a significant change in wear between the tested samples. The comparative EN-GJL-150 cast iron exhibited behavior typical of oxidative-adhesive wear. The large variation in mass loss (6.8 mg to 21.7 mg) and the variation in the friction coefficient (µ = 0.22–0.46) suggest periodic formation and delamination of oxide layers during the running-in process. When these layers fracture, abrasive particles form, accelerating wear. These results are consistent with studies [12,17,36].

In turn, the GJL-150 + SiC composite exhibited mild abrasive wear. The foam structure of the SiC insert appears to act as a “barrier,” preventing direct metal-to-metal contact and inhibiting adhesive wear. It should be emphasized that the heterogeneity of the tested materials limits the precision of the measurement method. This is particularly true for the countersample, which, due to its complex chemical composition, is macroscopically heterogeneous. Its components can cause excessive chipping of the friction lining. Therefore, it can be concluded that tribological testing should be performed on specific types of brake pads installed in motor vehicles.

It should be clearly noted that the advantage of the developed composites is not an increase in the coefficient of friction, but rather the small scatter of results (Figure 2b). This benefit may be a potential worth developing, as the small scatter of results is an advantage of the brakes themselves in terms of efficiency and uniform wear. To fully demonstrate the advantages of composite cast iron–ceramic brake discs over pure cast iron discs, a dedicated brake pad material for the developed composites would need to be identified.

The samples’ weight loss also reduced the weight of the entire brake disc casting. Prototype brake discs made from the developed composites reinforced with ceramic particles are lighter than discs made from cast iron alone. A brake disc of similar dimensions (∅ = 350 mm) made from GJL-150 gray cast iron weighs approximately 5800 g, while one made from composites weighs approximately 10% less (5220 g). The weight reduction results from the lower density of the porous structure (approx. 1 g cm^−3^) and the lower porosity of the foam structure itself (approx. 80%).

The second part of the study involved a comparison of microstructures. The results showed that traditional GJL-150 cast iron is characterized by a uniform distribution of “A”-type graphite (Figure 5a–d). Obtaining porous structures did not affect the uniformity of the microstructure of the tested samples. Within the cast iron, “A”-type graphite precipitates are still present and uniformly distributed in the pearlite matrix. However, it should be noted that research into the effects of SiC and Al_2_O_3_ particles on changes in the microstructural components of EN-GJL-150 cast iron should be continued and is currently in the phase of further experiments.

The introduction of a foreign phase, in the form of ceramic foam, into cast iron significantly alters the crystallization conditions. The composite production method presented in this study combines volumetric crystallization (on endogenous inclusions) and surface crystallization (on exogenous reinforcement). This leads to the formation of a unique microstructure in the transition zone—phase boundaries (Figure 8).

Aluminum oxide, a passive and stabilizing component, ensures the mechanical integrity of the porous structure (ceramic skeleton) at high temperatures. It is a material with high bond energy and relatively poor wettability by liquid cast iron (contact angle θ > 90° in nonreactive systems). Its role is to create a durable reinforcing system, typical of composites.

Silicon carbide acts as an active (reactive) component. At the casting temperatures of gray cast iron (1440–1450 °C), SiC particles are thermodynamically unstable in contact with locally unsaturated silicon and decompose according to the reaction [28]:

SiC_(S)_ + Fe_(L)_ → FeSi_(L)_ + C_(g)_ (1)

where

S—solid state;
L—liquid state;
g—graphite.

Reaction (1) is fundamental to the formation of microstructural components. The release of silicon and carbon atoms into the cast iron–ceramic transition zone (Figure 7b) results in local, strong supersaturation of these elements. Silicon, a strongly graphitizing element, increases the thermodynamic activity of carbon, promoting its precipitation as graphite. SiC, therefore, acts as an “in situ” modifier. This desirable phenomenon prevents the formation of hard, brittle iron carbides (cementite) in the joint zone.

**Figure 10:**
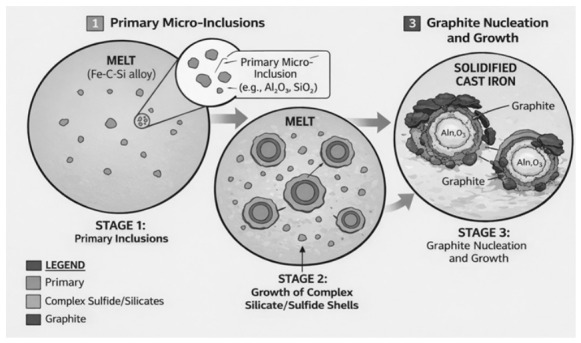
Illustrative diagram of graphite nucleation at the phase boundary: cast ironceramics in the EN-GJL-150 + SiC + Al_2_O_3_ composite.

Based on this, in the analyzed composites, Riposan’s theory [25,26,27] must be extended to include the influence of a macroscopic substrate in the form of reactive ceramic particles. This structure causes a non-uniform crystallization front with a geometry shaped by diffusion of the SiC particle surfaces. Nucleation on concave surfaces (e.g., cavities and gaps in the ceramic reinforcement) is energetically favored. The porous structure, with microcracks and surface cavities on the sintered SiC + Al_2_O_3_ particles, acts as a “geometric catalyst” for crystallization, promoting diffusion even under low-chemical-wettability conditions. A schematic diagram of graphite nucleation is shown in Figure 10.

**Figure 11:**
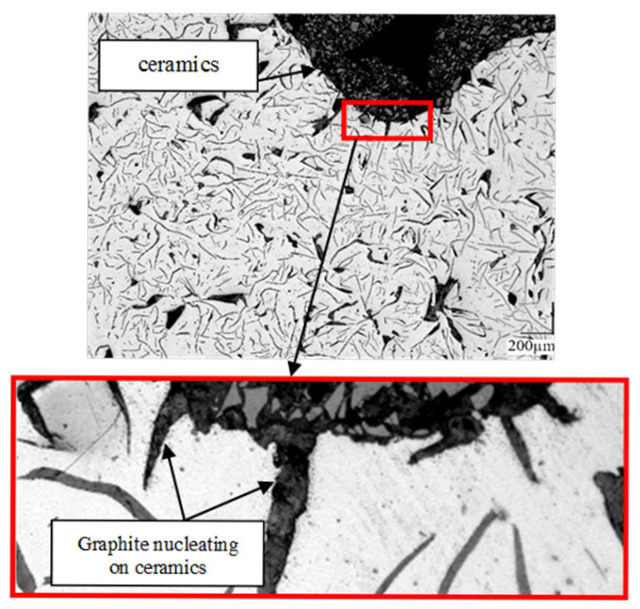
Microstructure of the EN-GJL-150 + SiC + Al_2_O_3_ composite containing graphite flakes nucleating on ceramic particles.

The proposed modification of Riposan’s theory is based on the fact that there are two coexisting processes of nucleation of graphite flakes (Figure 11):

1. Consistent with the Riposan model—deep within the metal matrix, away from the ceramic walls, graphite nucleates on (Mn, X)S sulfides, which in turn formed on (Al, Mg)XOX microoxides. The presence of MnS inclusions confirms this.
2. Surface-assisted nucleation (transition zone nucleation). At the phase boundary, the reinforcement surface, rich in oxides and carbides and highly developed topographically, takes over the role of the microoxides from the first stage. Sulfides of the (Mn, X)S type readily nucleate on the rough ceramic surface, forming an intermediate layer on which graphite grows directionally. This explains the very good adhesion of graphite to the ceramic and the absence of a graphite-free zone.

The presented results, although still preliminary, confirm that microstructure control through appropriately designed heat treatment plays a significant role in improving the functional properties of cast iron used in braking systems. Good results in increasing the damping and mechanical properties as well as HB (Brinell hardness) (hardness and consequently wear resistance are achieved by heat treatment with continuous cooling at a rate of 10 °C s^−1^ [44].

In the context of tribological applications, such microstructure modification can also contribute to increased wear resistance and friction stability of surface layers. A similar approach, based on microstructural modification to improve the mechanical and tribological properties of cast iron, represents an important research direction for the development of components subjected to high mechanical and thermal loads.

To strengthen the interpretation of the results, a more explicit correlation between microstructural features and tribological behavior should be emphasized. The improved wear resistance of the EN-GJL-150-based composites can be directly related to the modified microstructure. The uniform distribution of type A graphite flakes promotes the formation of a stable lubricating tribofilm during sliding, which reduces direct metal-to-metal contact and stabilizes the friction coefficient.

Furthermore, the presence of SiC and Al_2_O_3_ particles introduces a heterogeneous microstructure with a well-developed transition zone at the metal–ceramic interface. This region, enriched with (Mn, X)S inclusions and graphite nucleation sites, enhances load transfer and limits plastic deformation of the matrix. In particular, SiC particles, due to their partial reactivity and contribution to local carbon and silicon enrichment, promote graphitization and suppress cementite formation, leading to a more compliant and wear-resistant surface layer.

From a tribological perspective, these microstructural modifications result in a transition from oxidative–adhesive wear in the base cast iron to predominantly mild abrasive wear in the composites. The ceramic particles act as load-bearing elements and barriers to adhesion, while the graphite phase provides solid lubrication. The synergistic interaction between these constituents explains the reduced mass loss and improved stability of the friction coefficient observed for the SiC + Al_2_O_3_-reinforced composite.

To better position the obtained results within the current state of knowledge, a comparison with similar cast iron–ceramic composites reported in the literature is necessary. Previous studies on gray cast iron reinforced with ceramic particles such as SiC, Al_2_O_3_, TiC, or ZrO_2_ have consistently shown improvements in wear resistance due to the introduction of hard, load-bearing phases and modification of the microstructure. For example, composites reinforced with SiC particles typically exhibit a reduction in wear rate associated with increased hardness and the formation of protective tribolayers, while Al_2_O_3_-reinforced systems are known for their thermal stability and resistance to high-temperature degradation. However, in many reported cases, these systems suffer from increased brittleness or instability of the friction coefficient.

In comparison, the results obtained in this study show that the hybrid reinforcement (SiC + Al_2_O_3_) leads to a more favorable combination of properties, including a significant reduction in mass loss (approximately 6.6 times lower than that of unreinforced EN-GJL-150 cast iron) and a narrower, more stable friction coefficient range (µ ≈ 0.18–0.24). This behavior suggests a synergistic effect of the two ceramic phases, where SiC contributes to graphitization and interfacial reactivity, while Al_2_O_3_ stabilizes the structure and enhances mechanical integrity. Compared to literature data on monolithic cast iron or single-particle-reinforced composites, the developed material demonstrates improved wear resistance and friction stability, which are critical for brake disc applications. However, it should be noted that differences in testing conditions (e.g., load, sliding speed, counterface material) limit direct quantitative comparison, and further standardized studies are required to fully benchmark the performance of the proposed composite.

Table 5 summarizes a comparison between the obtained results and selected literature data on cast iron–ceramic composites. The developed hybrid composite exhibits competitive or improved tribological performance, particularly in terms of wear reduction and friction stability.

Observations of the microstructure of the EN-GJL-150 + SiC + Al_2_O_3_ composite after tribo-logical testing (Figure 9) indicate that titanium sulfides and carbides (carbide-nitrides) are present in the metallic phase. The TiC content was estimated at 0.25%. The sulfides present in cast iron contain titanium, forming complex sulfides of Mn, Fe, and Ti. No significant segregation of alloying elements was observed. Sulfides and carbides are evenly distributed throughout the matrix.

Research into the influence of SiC and Al_2_O_3_ particles on the microstructural composition of EN-GJL-150 cast iron, particularly graphite nucleation, is ongoing and should continue. It is also worth noting that the presented concept of obtaining composites through a porous structure containing ceramic SiC and Al_2_O_3_ particles has significant research potential for bonding cast iron with ceramics. At this stage of research, the material appears to meet its functional requirements, but further research is needed to assess its impact on other material properties, such as strength, fatigue, heat dissipation, and thermal shock, as well as to test actual brake discs under industrial conditions. Research in this area is ongoing.

It should be noted that, compared to traditional monolithic materials, the interfaces between individual phases play a significantly greater role in metal composite materials. The properties of the final product are directly influenced by the structure and properties of the interface between the reinforcing phase and the metal matrix, which depend on the technological parameters of production and operating conditions.

## 5. Conclusions

Based on the research results, the following conclusions were drawn:

1. The use of porous structures containing SiC and Al_2_O_3_ particles as a reinforcing phase simplifies the manufacturing process for composite brake discs, ensuring a uniform distribution of reinforcement without additional mixing. This reduces production costs and stabilizes tribological properties, resulting in a smaller spread of the friction coefficient.
2. The developed materials exhibit reduced abrasive wear. For the EN-GJL-150 + SiC composite, this reduction is approximately 4.9 times, for EN-GJL-150 + Al_2_O_3_—approximately 4.5 times, and for EN-GJL-150 + SiC + Al_2_O_3_—approximately 6.6 times compared to pure cast iron. This reduced abrasive wear stabilizes the required friction coefficient value over a run-in distance of less than 100 m. This may improve braking efficiency on so-called “cold brakes.”
3. The resulting narrow coefficient-of-friction range (0.18–0.28) enables the material to achieve the required cohesion under abrasion. This is one of the key criteria required by advanced stability control systems. Selecting a dedicated abrasive material can adjust the coefficient of friction to the appropriate level for the specific application.
4. The SiC + Al_2_O_3_ reinforcement particles act as active catalysts for graphite nucleation. This eliminates the unfavorable cementite precipitates in the transition zone, which have previously been a problem in cast iron-based composite materials. This structural cohesion at the cast iron–ceramic interface is a direct result of the favorable crystallographic match between the ceramic and the pearlitic matrix.
5. SEM images show a clear interface between the cast iron and the porous ceramic structure. No cracks, delaminations, or gas pores were observed, which are common in in situ cast composites. This indicates the correct selection of pouring parameters and adequate wettability of the ceramic by the molten metal. It is worth noting that with a 70% SiC content, the ceramic is reactive, which favors chemical wetting.
6. Analysis of the graphite and matrix morphology in the transition zone and the traditional matrix reveals 100% pearlite precipitates. No fragmented or degenerated graphite was observed, which is a common problem when cast iron is in contact with a ceramic reinforcing phase. However, homogeneously distributed type A flake graphite is visible.
7. The expansion of graphite flakes from the surface of ceramic particles (Figure 11) suggests that the reinforcement surface “took over” the function of a heterogeneous nucleus.
8. The presence of manganese and sulfur in the transition zone indicates that the ceramic surface can act as a substrate for sulfide nucleation (replacing microoxides formed in the first stage of the nucleation process), thereby initiating graphite growth. This demonstrates the synergistic action of endogenous and exogenous nucleation processes. The reaction of cast iron with SiC created a local zone rich in carbon and silicon, significantly increasing the graphitization potential. The manganese and sulfur present in the (Mn, X)S sulfides at the cast iron–ceramic interface are the primary changes that support Riposan’s model.

## Figures and Tables

**Table 1:** Results of the chemical composition of EN-GJL-150 cast iron.

Element Content, wt.% *						
C	Si	Mn	P	S	Fe	Other *
3.72	1.65	0.66	0.03	0.067	93.5–94.0	Al; Sr; Zr

*—up to approximately 0.005 wt.% Al; up to 0.008 wt.% Sr, up to 0.006% Zr. These are the elements contained in the Elkem SuperSeed modifier.

**Table 2:** Tribological test results—sample mass loss, ∆m GJL.

Sample Designation	Measurement No.					Average Value, mg	Δm GJL (Max−Min), mg	Relative Mass Consumption Compared to Cast Iron, %
	1	2	3	4	5			
GJL-150 + SiC	4.0	2.2	3.5	3.8	1.6	3.01	2.40	20.47
GJL-150 + Al_2_O_3_	4.0	3.2	2.3	3.5	3.6	3.30	1.70	22.44
GJL-150 + SiC + Al_2_O_3_	1.8	2.2	2.7	2.4	2.1	2.23	0.90	15.17
GJL-150	6.8	19.6	15.6	9.8	21.7	14.70	14.90	100

**Table 3:** Tribological test results—counter-sample mass loss, ∆m P.

Sample Designation	Measurement No.					Average Value, mg	Δm P (Max−Min), mg
	1	2	3	4	5		
GJL-150 + SiC	2.8	2.4	3.3	3.2	2.3	2.81	1.00
GJL-150 + Al_2_O_3_	5.0	4.4	4.6	4.8	4.2	4.61	0.80
GJL-150 + SiC + Al_2_O_3_	3.3	3.4	5.8	4.0	4.3	4.16	2.50
GJL-150	3.0	5.7	8.2	9.9	12.7	7.91	9.70

**Table 4:** Tribological test results—friction coefficient μ.

Sample Designation	Measurement No.					Average Value
	1	2	3	4	5	
GJL-150 + SiC	0.20–0.26	0.20–0.26	0.22–0.26	0.24–0.28	0.22–0.26	0.220–0.280
GJL-150 + Al_2_O_3_	0.24–0.26	0.21–0.23	0.24–0.28	0.22–0.24	0.23–0.25	0.225–0.255
GJL-150 + SiC + Al_2_O_3_	0.18–0.22	0.20–0.24	0.22–0.26	0.20–0.24	0.20–0.24	0.200–0.240
GJL-150	0.16–0.32	0.26–0.42	0.24–0.40	0.18–0.34	0.22–0.46	0.220–0.390

**Table 5:** Comparison of tribological properties of EN-GJL-150-based composites with literature data.

Material System	Reinforcement	Test Conditions (Approx.)	Wear Rate/Mass Loss	Friction Coefficient (µ)	Key Observation	References
EN-GJL-150 (base)	None	Pin-on-disc, dry	14.7 mg/5000 m	0.22–0.46	High wear, unstable µ	This work
EN-GJL-150 + SiC	10% SiC	Same as above	3.01 mg/5000 m	0.22–0.28	Reduced wear, mild abrasive mechanism	This work
EN-GJL-150 + Al_2_O_3_	10% Al_2_O_3_	Same as above	3.30 mg/5000 m	0.22–0.25	Stable µ, higher counter-sample wear	This work
EN-GJL-150 + SiC + Al_2_O_3_	Hybrid (70/30)	Same as above	2.23 mg/5000 m	0.18–0.24	Lowest wear, highest stability	This work
Gray cast iron + SiC	5–15% SiC	Dry sliding	↓ wear (3–5× reduction)	0.20–0.30	Increased hardness, abrasive wear dominates	[28,30]
Cast iron + Al_2_O_3_	~10% Al_2_O_3_	Dry sliding	Moderate wear reduction	0.22–0.28	Improved thermal stability, brittle behavior possible	[32]
Cast iron + TiC	5–10% TiC	Dry sliding	Significant wear reduction	0.25–0.35	High hardness, risk of brittleness	[30]
Cast iron + ZrO_2_	~10% ZrO_2_	Dry sliding	Improved wear resistance	0.20–0.27	Good thermal resistance, stable µ	[31]
Conventional brake disc (gray cast iron)	None	Industrial conditions	High wear variability	0.30–0.45	Oxidative–adhesive wear dominates	[12,17,36]

## Data Availability

The original contributions presented in this study are included in the article. Further inquiries can be directed to the corresponding author.
